# Home oxygen use and 1-year outcome among preterm infants with bronchopulmonary dysplasia discharged from a Chinese regional NICU

**DOI:** 10.3389/fped.2022.978743

**Published:** 2022-09-09

**Authors:** Huijia Lin, Xuefeng Chen, Jiajing Ge, Liping Shi, Lizhong Du, Xiaolu Ma

**Affiliations:** ^1^Department of NICU, The Children’s Hospital, Zhejiang University School of Medicine, National Clinical Research Center for Child Health, Hangzhou, China; ^2^Department of Endocrinology, The Children’s Hospital, Zhejiang University School of Medicine, National Clinical Research Center for Child Health, Hangzhou, China

**Keywords:** home oxygen, bronchopulmonary dysplasia, preterm infants, readmission, follow-up

## Abstract

**Objective:**

This study aims to compare the clinical characteristics and 1-year outcomes of preterm infants with bronchopulmonary dysplasia (BPD) who were discharged on supplemental oxygen or room air.

**Materials and Methods:**

The preterm infants (born <32 weeks’ gestation, birth weight ≤1,250 g) diagnosed with BPD and admitted between January 2020 and December 2020 were enrolled. The clinical data during hospitalization were collected through the hospital’s electronic record system. The outcomes after discharge were acquired from the outpatient system and through telephonic interviews.

**Results:**

Of the 87 preterm infants diagnosed with BPD, 81 infants survived until discharge. The 81 infants were divided into the home oxygen group (*n* = 29) and room air group (*n* = 52) according to supplemental oxygen or not at discharge. Infants in the home oxygen group were more likely to receive postnatal systemic steroids and higher ventilation settings at 36 weeks’ PMA. There was one patient in each group who died before 1 year corrected age, respectively. All the infants had successfully weaned off oxygen eventually during the first year. The median duration of home oxygen therapy was 25 (7,42) days. Readmission occurred in 49 (64.5%) infants. Readmissions for infants with home oxygen were more often related to respiratory disease. In addition, wheezing disorders and home inhalation occurred more frequently in the home oxygen group (*p* = 0.022, *p* = 0.004). Although the incidence of underweight at 1 year corrected age was higher in the room air group (10.0 vs. 3.8%), there was no significant difference (*p* = 0.620). The rate of neurodevelopmental impairment was similar between these two groups (26.0 vs. 30.8%, *p* = 0.659).

**Conclusions:**

It was the first study focused on preterm infants with BPD receiving home oxygen in China. Infants with home oxygen were more likely to have respiratory problems after discharge from NICU. Home oxygen use was not associated with more readmission for infants with BPD, and no difference was found in neurodevelopmental impairment and growth outcome.

## Introduction

In recent decades, with the development of perinatal and neonatal care in China, more and more preterm infants have been treated and survived (1). However, the lung development of these infants is susceptible to various injuries, resulting in bronchopulmonary dysplasia (BPD) (2). The incidence of bronchopulmonary dysplasia (BPD) was also increasing during these decades in China (3, 4).

Bronchopulmonary dysplasia is one of the most common and complex complications for preterm infants that brings heavy burden to the families and the healthcare system. Also, BPD is related to prolonged hospital stay and comorbidity. In a developed country, home oxygen supplementation is a common post-discharge therapy for infants with chronic BPD (5). It could shorten hospital stay significantly, save medical resource, and prevent prolonged separation from the family (6). In recent years, some parents in our country have gradually accepted home oxygen therapy for their chronic BPD infants. However, we are still lacking general consensus regarding home oxygen for BPD infants in China. Moreover, there are less actual data about long-term follow-up and outcome for these infants with home oxygen post-discharge.

Therefore, we designed this study to investigate the clinical characteristics and long-term follow-up till 1 year corrected age in preterm infants diagnosed with BPD based on 2018 NICHD criteria who were discharged on supplemental oxygen from our NICU compared with BPD infants discharged in room air. We hypothesized that home oxygen use would be associated with more complications and more frequent readmissions during the first year.

## Materials and methods

### Study design and patients

We performed a retrospective cohort study using data from the Department of Neonatology, Children’s Hospital, Zhejiang University School of Medicine. This is a 50-bed NICU with around 1,000 annual admissions. The inclusion criteria were as follows: (1) Preterm infants whose gestational age (GA) was <32 weeks and whose birth weight (BW) was ≤1,250 g were admitted from January 2020 to December 2020; and (2) preterm infants diagnosed with BPD according to the 2018 NICHD consensus. Exclusion criteria were as follows: (1) Major congenital anomalies, including serious congenital heart defects, chromosomal abnormalities, brain malformations, congenital diaphragmatic hernia, and anomalies of the digestive tract or kidney; (2) inborn errors of metabolism or severe heritable disease; and (3) died during hospitalization. The enrolled infants were divided into the home oxygen group and room air group based on the need for oxygen supplementation or room air at discharge.

### Implementation of home oxygen

The preterm infants with low flow nasal oxygen would have room air test predischarge. If the infants’ oxygen saturations remained >92% in room air over 30 min, they were counted as passing the test. Conversely, it was counted as failing the test and couldn’t wean off oxygen. Home oxygen was defined as infants with BPD who couldn’t wean off oxygen successfully and still needs flow oxygen to maintain saturation between 92 and 95% (7) at discharge.

A discharge education team was composed of nurse practitioners and neonatal fellows. As the infants’ situation is close to discharge, discharge education including feeding skills, medication information, and other nursing skill will be arranged. Usually, it takes 2 weeks for parents to be comfortable with taking their preterm infant home. If the parents were confident with discharge home on oxygen supplementation, the education team will give additional education including the use of an oxygenator, the saturation monitor, how to evaluate the infant, and so on. It takes at least 1 week to get all the equipment and knowledge prepared. We also have videos and brochures for parents’ review at home.

### Definitions of the relevant concepts

The BPD was diagnosed and graded according to the 2018 NICHD consensus (8). Intraventricular hemorrhage (IVH) was defined according to Papile’s classification. Severe IVH was defined as greater than or equal to grade 3. Periventricular leukomalacia (PVL) was defined as the presence of periventricular cysts identified on cranial ultrasonography or magnetic resonance imaging. Necrotizing enterocolitis (NEC) was defined as greater than stage 2 based on the modified Bell’s staging (9). Retinopathy of prematurity (ROP) and its graded standard were determined based on the international classification of ROP. Small for gestational age (SGA) and extrauterine growth retardation (EUGR) were described as infants whose weight was less than the 10th percentile for corrected gestational age, according to the Fenton intrauterine growth curves. Hemodynamically significant patent ductus arteriosus (hsPDA) was defined as when the ductus diameter exceeds 1.5 mm, left atrial inner diameter/aortic root (LA/AO) exceeds 1.4, and combines left-to-right shunt by echocardiography (10). Pulmonary hypertension associated with BPD (BPD-PH) was diagnosed by echocardiography. The criteria for BPD-PH were met by any of the following findings (11, 12): (1) systolic pulmonary artery pressure (sPAP)/systolic blood pressure(sBP) >0.5; (2) Cardiac shunt with bidirectional or right-to-left flow; and (3) If no tricuspid regurgitation shunt, the following criteria were recorded as PH, including right atrial enlargement, right ventricular dilation, right ventricular hypertrophy, and ventricular septal flattening. Weight and length at 1 year corrected age were calculated with WHO Child Growth Standards (13). Underweight was defined as >2 SD below the mean weight for age. Wheezing disorders were diagnosed of wheezing exposure treated with anti-asthma medicines (bronchodilators and corticosteroids) (14). Neurodevelopmental assessment at 12 months corrected age was performed by Bayley Scales of Infant Development, version 2 (Bayley-2), including the Mental Developmental Index (MDI) and Psychomotor Developmental Index (PDI). Neurodevelopment delay was defined as MDI < 70 or PDI < 70 (15, 16). Cerebral palsy (CP) refers to a non-progressive disability of movement and posture and was diagnosed on the basis of abnormal muscle tone and reflexes on the physical and neurological examination (17). Neurodevelopment impairment included MDI < 70, PDI < 70, and CP. This study was approved by the Ethics Committee of the Children’s Hospital of Zhejiang University School of Medicine (Ethics No. 2022-IRB-141).

### Data collection

The data regarding hospitalization were retrospectively collected from patient charts or electronic systems, including demographics, perinatal factors, maternal history, management or treatment, and morbidity.

All surviving infants were routinely followed up and underwent developmental assessment in the clinic at 3, 6, 9, and 12 months corrected age. The development outcome and readmission during discharge to 1 year corrected age were collected from outpatient electronic records system or by telephonic interviews.

### Statistical analysis

All clinical data were statistically analyzed using SPSS software version 20.0. Variables without normal distributions were analyzed using the Wilcoxon-Mann-Whitney test and were presented as median [interquartile ranges (IQRs)]. Continuous variables with normal distributions were summarized as the mean ± standard deviation (SD). Categorical variables were presented as numbers (%) of subjects. Student’s *t*-test was examined through the comparisons of continuous variables. The comparisons of categorical variables were analyzed using the Pearson’s chi-square test or Fisher’s exact test. *P* < 0.05 was considered statistically significant.

## Results

### Patients and characteristics

**FIGURE 1:**
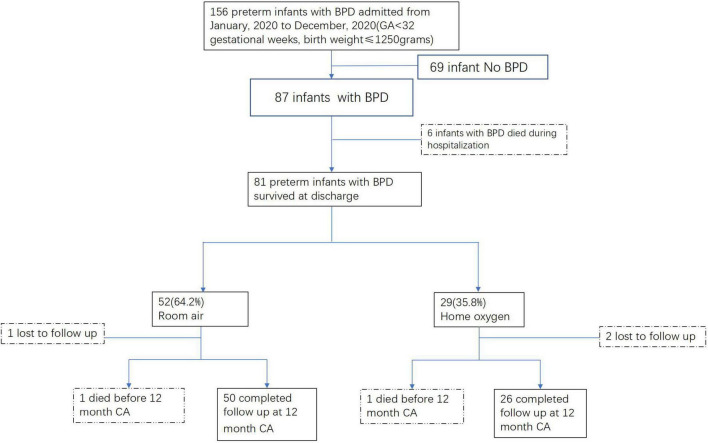
Study flow.

Totally, there were 156 preterm infants with GA < 32 weeks and BW ≤ 1,250 g admitted to our unit from January 2020 to December 2020, 87 of whom were diagnosed with BPD. Except 6 infants died during hospitalization, 81 infants survived at discharge; 29 (35.8%) infants went home with supplemental oxygen, and the other 52 (64.2%) infants were in room air at discharge (Figure 1).

The mean GA was 28 weeks, and the mean BW was 1,050 g for the survived BPD infants. The GA, BW, male sex proportion, date at admission, multiple births, assisted reproduction, and Apgar score at 5 min were similar between the home oxygen group and room air group. For maternal characteristics, the age, education level, and complications (diabetes, hypertension) of the mother were similar for the two groups. The incidence of comorbidity including IVH, severe IVH, PVL, ROP with surgery, NEC, and hsPDA was not significantly different between the two groups. In terms of treatment, infants in the home oxygen group were more likely to receive postnatal systemic steroids and higher ventilation settings (MAP × FiO_2_) at 36 weeks’ PMA (*p* = 0.018, *p* = 0.026). But the ventilation days and BPD severity were no significant difference between the two groups. With regard to growth in the hospital, although the weight, length, and head circumference at discharge were similar for these infants in the two groups, the incidence of EUGR at discharge was a bit lower in the home oxygen group (31.0 vs. 53.8%, *p* = 0.048). The clinical and demographic data of these patients are presented in Table 1.

**TABLE 1:** Clinical and demographic characteristics of preterm infants with BPD in two groups.

	All	Room air	Home oxygen	Z/*t/χ^2^*	*P*-value
*N* (%)	81	52 (64.2)	29 (35.8)	–	–
GA, weeks, median (IQR)	28.0 (27.2,29.1)	28.0 (26.9,29.1)	28.0 (27.4,29.2)	−0.523	0.601
BW, grams, median (IQR)	1050 (950,1180)	1070 (913,1168)	1035 (970,1240)	−0.355	0.723
Date at admission, day, median (IQR)	9 (1,25)	10 (1,23)	8 (1,27)	−0.408	0.684
SGA, *n*/*N* (%)	4/81 (4.9)	2/52 (3.8)	2/29 (6.9)	–	0.615
Male sex, *n*/*N* (%)	50/81 (61.7)	33/52 (63.5)	17/29 (58.6)	0.185	0.667
Apgar score at 5 min, score, median (IQR)	9 (8,10)	9 (8,10)	9 (8,10)	−1.1444	0.149
Maternal diabetes, *n*/*N* (%)	8/81 (9.9)	5/52 (9.6)	3/29 (10.3)	0.011	0.916
Mother’s age, year, median (IQR)	30 (27,36)	30 (27,35)	29 (26,36)	0.785	0.435
Maternal hypertension, *n*/*N* (%)	14/81 (17.3)	9/52 (17.3)	5/29 (17.2)	0.000	0.994
Maternal education more than high school^$^, *n*/*N* (%)	40/61 (65.6)	23/37 (62.2)	17/24 (70.8)	0.485	0.486
Multiple births, *n*/*N* (%)	30/81 (37.0)	20/52 (38.5)	10/29 (34.5)	0.126	0.722
Assisted reproduction, *n*/*N* (%)	25/81 (30.9)	16/52 (30.8)	9/29 (31.0)	0.001	0.980
BPD Severity, *n*/*N* (%)					
Grade 1	44/81 (54.3)	29/52 (55.8)	15/29 (51.7)	0.123	0.726
Grade 2	19/81 (23.5)	13/52 (25.0)	6/29 (20.7)	0.193	0.661
Grade 3	18/81 (22.2)	10/52 (19.2)	8/29 (27.6)	0.752	0.386
Ventilator days, days, median (IQR)	9 (3,20)	9 (3,18)	10 (3,32)	−0.609	0.543
MAP × FiO2@36 weeks’ PMA for ventilated patients, median (IQR)	4.5 (3.7,7.0)	4.2 (3.5,4.2)	6.9 (6.4,7.6)	−2.374	0.018*
Systemic steroids, *n*/*N* (%)	16/81 (19.8)	7/52 (13.5)	10/29 (34.5)	4.961	0.026*
hsPDA ligation, *n*/*N* (%)	33/81 (40.7)	22/52 (42.3)	11/29 (37.9)	0.148	0.701
Drug therapy for HsPDA, *n*/*N* (%)	26/81 (32.1)	15/52 (28.8)	11/29 (37.9)	0.705	0.401
NEC, *n*/*N* (%)	12/81 (14.8)	11/52 (21.2)	1/29 (3.4)	3.328	0.068
Surgical NEC, *n*/*N* (%)	8/81 (9.9)	7/52 (13.5)	1/29 (3.4)	1.123	0.289
IVH, *n*/*N* (%)	40/81 (49.4)	27/52 (51.9)	13/29 (44.8)	0.375	0.540
Severe IVH, *n*/*N* (%)	9/81 (11.1)	7/52 (13.5)	2/29 (6.9)	0.812	0.367
Surgical ROP, *n*/*N* (%)	10/81 (12.3)	7/52 (13.5)	3/29 (10.3)	0.003	0.955
BPD-PH at discharge, *n*/*N* (%)	5/81 (6.2)	2/52 (3.8)	3/29 (10.3)	–	0.609
PVL, *n*/*N* (%)	11/81 (13.6)	9/52 (17.3)	2/29 (6.9)	0.947	0.331
PMA at discharge home, weeks, median (IQR)	39.6 (38.4,41.9)	39.7 (38.3,41.8)	39.1 (38.3,42.2)	−0.039	0.969
LOS, days, median (IQR)	67 (53,86)	66 (52,86)	67 (55,92)	−0.419	0.675
EUGR at discharge, *n*/*N* (%)	37/81 (45.7)	28/52 (53.8)	9/29 (31.0)	3.904	0.048*
Weight at discharge, kilogram, median (IQR)	2.95 (2.58,3.5)	2.9 (2.5,3.4)	3.0 (2.69,3.59)	−1.461	0.144
Length at discharge, centimeter, median (IQR)	49.5 (46.0,50.0)	50.0 (46.0,50.0)	50.0 (46.3,50.0)	−0.352	0.725
HC at discharge, centimeter, median (IQR)	33.0 (31.5,34.5)	33.0 (31.5,34.4)	33.0 (32.0,34.9)	−0.748	0.455

GA, gestational age; BW, birth weight; SGA, small for gestational age; BPD, bronchopulmonary dysplasia; MAP, mean airway pressure; PDA, patent ductus arteriosus; NEC, necrotizing enterocolitis; IVH, intraventricular hemorrhage; ROP, retinopathy of prematurity; LOS, length of stay; PVL, periventricular leukomalacia; PMA, postmenstrual age; EUGR, extrauterine growth retardation; HC, head circumference; PH: pulmonary hypertension; IQR, interquartile range. ^$^Room air group n = 37, Home oxygen n = 24. *P < 0.05.

### Follow-up and home oxygen

During the first year after discharge, 1 infant in the room air group and 2 infants in the home oxygen group were lost to follow-up. One patient in each of the two groups died before 1 year corrected age, respectively. The infant in the room air group died at 2 months corrected age due to asphyxia. The infant with home oxygen died at 11 months corrected age due to severe pneumonia and ventricular tachycardia in the pediatric ICU. The mortality between the two groups had no significant difference. Thus, there were 76 infants who received long-term follow-up (Figure 1).

Totally, there were 5 (6.2%) infants diagnosed with BPD-PH at discharge. Although the rate of PH was a bit higher in the home oxygen group than that in the room air group (10.3 vs. 3.8%), there was no significant difference between the two groups. Fortunately, these 5 infants had relieved before 1 year corrected age. Of the 76 infants, 31 (40.8%) infants had irregular follow-up clinic visits, and the other 45 (59.2%) infants had regular and complete follow-up. The reason for irregular visits was investigated by telephonic interviews: 13 (17.1%) infants due to the impact of COVID-19, and other 18 (23.7%) due to far distance from our hospital and got clinic visits at the local hospital.

For the preterm infants with oxygen supplementation, the mean flow and fraction of inspired O_2_ (FiO_2_) was 1.0 (1.0, 1.0) liter per minute, 25% (23, 25%) at discharge. After discharge, continuous oxygen was used in 17 (65.4%) cases, and intermittent oxygen was used in other 9 (34.6%) cases. Among these infants with home oxygen, 11 (42.3%) cases were under the direct guidance of a pediatrician or neonatologist for ceasing oxygen, and 15 (57.7%) infants weaned off oxygen mainly based on parents’ decisions or indirect guidance from a physician using internet or telephone. Fortunately, all the infants had successfully weaned off oxygen eventually during the first year. The median duration of home oxygen therapy was 25 (7,42) days.

### Readmissions

During the first year, 49 (64.5%) infants were readmitted to the hospital at least once before 1 year corrected age. The median frequency of readmissions for these 49 infants was 2 (1,3) times. The median total days of readmission duration were 13 (7,21) days. The interval of the first readmission after discharge was 51 (15,149) days in the room air group and 13 (6,36) days in the home oxygen group, respectively, but there was no significant difference between the two groups (*p* = 0.069). The odds, frequency, and duration of readmission were not significantly different between these two groups. But readmissions for infants with home oxygen were more often related to respiratory disease (*p* = 0.007). In addition, wheezing disorders occurred more frequently in the home oxygen group (80.8 vs. 54.0%, *p* = 0.022). Therefore, home inhalation therapy was used more for infants with home oxygen (57.7 vs. 24.0%, *p* = 0.004). Totally, there were 12 infants who had received different kinds of surgery, including ROP, inguinal hernia, and fistula closure. The rate of surgery was similar in the two groups. All the data on readmission outcomes are presented in Table 2.

**TABLE 2:** Readmission outcome between the two groups.

	All	Room air	Home oxygen	Z/*t/χ^2^*	*P*
*N*, (%)	76	50	26	–	–
Mortality, *n*/*N* (%)	2/76 (2.6)	1/50 (2.0)	1/26 (3.7)	–	1.000
Readmission, *n*/*N* (%)	49/76 (64.5)	31/50 (62.0)	19/26 (73.1)	0.933	0.334
Total readmission duration, days, median (IQR)	13 (7,21)	13 (7,22)	12 (7,21)	−0.492	0.623
Readmission frequency, median (IQR)	2 (1,3)	2 (1,3)	1 (1,3)	−1.154	0.248
Interval of first readmission after discharge, days, median (IQR)	35 (12,132)	51 (15,149)	13 (6,36)	−1.821	0.069
Proportion of readmission for respiratory disease,%, median (IQR)	100 (50,100)	50 (0,100)	100 (67,100)	−2.687	0.007*
Proportion of readmission for non-respiratory disease, %, median (IQR)	0 (0,50)	50 (0,100)	0 (0,33)	−2.687	0.007*
Wheezing disorders, *n*/*N* (%)	48/76 (63.2)	27/50 (54.0)	21/26 (80.8)	5.268	0.022*
Home inhalation, *n*/*N* (%)	27 (35.5)	12/50 (24.0)	15/26 (57.7)	8.477	0.004*
Surgery, *n*/*N* (%)	12/76 (15.8)	9/50 (18.0)	3/26 (11.5)	0.161	0.688

*P < 0.05.

### Long-term outcomes

At 1 year corrected age, the weight and length were 9.0 (8.5,10.0) kg and 74.0 (73.0–75.6) cm, respectively. These were similar in the two groups. The proportion of underweight at 1 year corrected age was 10.0% in the room air group, which was higher than that in the home oxygen group, but there was no significant difference between the two groups (*p* = 0.620).

Regarding neurodevelopmental outcomes, 6 (7.9%) of 76 infants had cerebral palsy, and 21 (27.6%) and 11 (14.5%) infants had MDI and PDI less than 70. Totally, 13 (26.0%) infants in the room air and 8 (30.8%) infants in the home oxygen group were defined as having neurodevelopmental impairment. However, the incidence of neurodevelopmental impairment was similar between these two groups (*P* = 0.695) (Table 3).

**TABLE 3:** Long-term outcomes between the two groups.

	All	Room air	Home oxygen	Z/*t/χ^2^*	*P*
*N*, (%)	76	50	26	–	–
Weight at 1 year, kilogram, median (IQR)	9.0 (8.5,10.0)	9.0 (8.5,10.0)	9.4 (8.4,10.1)	−0.401	0.688
Length at 1 year, centimeter, median (IQR)	74.0 (73.0,76.0)	74.0 (73,75.6)	74.5 (72.0,76.5)	−0.176	0.860
Underweight, *n*/*N* (%)	6/76 (7.9)	5/50 (10.0)	1/26 (3.8)	0.246	0.620
PDI < 70 *n*/*N* (%)	21/76 (27.6)	13/50 (26.0)	8/26 (30.8)	0.195	0.659
MDI < 70, *n*/*N* (%)	11/76 (14.5)	7/50 (14.0)	4/26 (15.4)	0.026	0.871
Cerebral palsy, *n*/*N* (%)	6/76 (7.9)	4/50 (8.0)	2/26 (7.7)	0.000	1.000
Neurodevelopment impairment, *n*/*N* (%)	21/76 (27.6)	13/50 (26.0)	8/26 (30.8)	0.195	0.659

PDI, psychomotor development index; MDI, metal development index.

## Discussion

The BPD is a prevalent respiratory complication of preterm infants. Infants with BPD often require low concentrations of supplemental oxygen for prolonged periods, but are otherwise in good clinical condition (growing and feeding well and no longer requiring skilled nursing care) (18). Thus, home oxygen supplementation is a common therapy for infants with BPD upon NICU discharge in developed countries (7, 19). It was usually considered to shorten the hospital stay, which was confirmed by prior studies (20–22).

Actually, in recent years, home oxygen for BPD infants has been gradually applied in China. In the past, parents could not accept home oxygen therapy, partly due to the cost of equipment, or difficulty of taking care of preterm infants at home, or anxiety and fear. Therefore, even if the infants reach the standard of discharge with oxygen, parents still want to stay longer to attempt to wean oxygen in NICU. Thus, earlier intervention and education to facilitate parent confidence with infant care and medical equipment might be effective in safely reducing NICU length of stay for infants with BPD (23). With the promotion of kangaroo care (24), family integrated (FI) care (25), and other measures, more parents learn how to care for preterm infants during hospitalization and dare to take infants home with oxygen.

The rate of home oxygen for infants with BPD varies between different countries and regions. Ejiawoko et al. (20) reported that totally, 47% of infants with BPD were discharged with home oxygen in California and home oxygen use ranged from 7 to 95% among different level hospitals. Lagatta et al. (26) reported that home oxygen was used in 53% of infants in 22 regional NICUs in the United States. Kim et al. (27) found that the proportion of home oxygen was 13.7% in total VLBW infants and 54.6% in GA less than 29 weeks. Our single-center data showed that about one-third of infants with BPD needed home oxygen post-discharge.

Previous studies showed that infants with BPD who were discharged with home oxygen were more likely to be born at an earlier gestational age, be SGA, receive antenatal steroids, have more ventilator days, have a higher grade of BPD severity, and receive a PDA ligation (20, 26, 28, 29). However, our data showed that the GA, BW, BPD severity, ventilator days, and BPD grade were similar between home oxygen and room air group. It was also unexplainable that the proportion of EUGR at discharge was higher in the room air group although the *P*-value was critical. It might relate to sample size and the fewer proportion of younger GA (less than 26 weeks) in our study.

On the contrary, similar to some studies (26, 30), our present data also indicated that infants with home oxygen had higher ventilation settings at 36 weeks of PMA and were more likely to have postnatal systemic steroids. Therefore, our results confirmed that the infants with home oxygen had more severe BPD than infants with room air.

Our study showed that the duration of home oxygen use was 25 (7,42) days, which is shorter than previous studies (20, 27). Kim et al. reported that the duration of home oxygen was 69 ± 99 days for very low birth weight infants; however, Ejiawoko et al. showed that the median age of weaning off supplemental oxygen was 10.1 months after initial hospital discharge. It altered from different studies. The shorter duration of home oxygen in our study might be related to the low FiO2 and oxygen flow at initial discharge. Some parents were confident in taking care of their infants after adequate training and education and hoped to take infants home earlier. Actually, these preterm infants were not severe, so the duration of oxygen use was relatively short.

Due to different causes, 57.7% of infants weaning off oxygen were not face-to-face guided by physicians. Yeh et al. also reported that unsupervised weaning of supplemental oxygen occurred in 32.1% of infants with BPD from a single center in the United States. Usually, in developed countries, weaning oxygen for BPD infants was assessed for overnight polysomnography or evaluation in the BPD clinic during a regular appointment (31). If weaning off oxygen is not appropriate, inadequate oxygenation may impair brain development and somatic growth as well as alter cardiac function (32). For these infants who cannot receive face-to-face direction, there might be other supplemental measures, including internet hospital, telephone, or video link. Therefore, it is necessary to establish more standardized consensus or guidelines about weaning oxygen to guide parents in China.

Previous studies revealed that BPD increased the frequency of readmission, hospitalization, ICU admission rate and duration, and healthcare burden (33, 34). Readmission was a common problem for infants with BPD (35), which may be rehospitalized for various diseases including respiratory problems and non-respiratory diseases. Our results showed that home oxygen for infants with BPD was not associated with odds of readmission, which is similar to other studies (26, 36). We also found that infants with home oxygen were more likely to be rehospitalized for respiratory illness (36) and not for non-respiratory disease.

Preterm infants with BPD were thought to have higher rates of growth failure and neurodevelopmental disability when compared to preterm infants without BPD (37, 38). BPD was associated with chronic inflammation and recurrent hypoxemia, which negatively impact brain development and function (39). As home oxygen was commonly used in infants with BPD, which was thought to prevent hypoxemia and to allow for adequate growth, some studies focused on the development of infants with supplemental oxygen. But, the results were different among studies. A recent large cohort study compared 1,039 infants with moderate or severe BPD who were discharged with supplemental oxygen, as compared to propensity-matched infants discharged breathing room air. It showed that infants treated with supplemental oxygen at home had marginally improved growth but no improvement in developmental outcomes at 2 years corrected age (36). Another large longitudinal study compared 1,030 preterm infants of BW ≤ 1,250 g with no BPD, BPD, and BPD with chronic oxygen dependency and found that the growth outcome and neurodevelopmental disability were not different between BPD and BPD with chronic oxygen dependency at 3 years corrected age (30). Similar to previous studies, we suggested that the need for supplemental home oxygen in infants with BPD does not predict increased neurodevelopmental disability and growth delay compared to infants with BPD in room air. The long-term outcomes would be associated with multifactors, not only supplemental oxygen need.

To sum up, home oxygen could decrease hospital stay, reduce costs, and save NICU resources. It was safe and does not increase mortality or life-threatening event from our data. In future, standardized protocol needs to be established to guide follow-up and wean off oxygen for BPD infants with home oxygen.

The limitations of this study were as follows: (1) It was a retrospective study, in which details cannot be obtained if not recorded in the medical system, such as the baseline of oxygen saturation before wean off at home, or other unmeasured characteristics of illness. (2) The study enrolled a size limit of infants. (3) It was a single-center study from China. Our unit is a regional NICU, which represents the level of a tertiary pediatric center in eastern China. But there might be some differences in the treatment of premature infants from other parts of China. Further studies designed as prospective, multicenter research may lead to resolve these limitations. Despite these limitations, our study provides the clinical characteristics and long-term living conditions for BPD infants with home oxygen from China.

## Conclusion

It was the first study to focus on preterm infants with BPD receiving home oxygen in China. Infants with BPD in home oxygen were more likely to have respiratory problems after discharge from NICU. Home oxygen use was not associated with readmission for infants with BPD, and also, no difference was found in the neurodevelopmental impairment and growth outcome. It might need a standardized protocol to guide follow-up and wean off oxygen for BPD infants with home oxygen in China.

## Data availability statement

The original contributions presented in this study are included in the article, further inquiries can be directed to the corresponding author.

## Ethics statement

The studies involving human participants were reviewed and approved by the Ethics Committee of the Children’s Hospital of Zhejiang University School of Medicine. Written informed consent from the participants’ legal guardian/next of kin was not required to participate in this study in accordance with the national legislation and the institutional requirements.

## Author contributions

HJL participated in the design of the study, collected clinical data and telephone follow-up, provided interpretation of data, and drafted the initial manuscript. XFC conducted the statistical analyses. JJG collected clinical data together. LPS and LZD conducted the study. XLM conceptualized and supervised the design and execution of the study, reviewed the analyses, and critically reviewed and revised the manuscript. All authors contributed to the article and approved the submitted version.
